# The Impact of International Board Certified Lactation Consultant (IBCLC) Support on Breastfeeding in the UK and Ireland—A Scoping Review

**DOI:** 10.1111/mcn.70189

**Published:** 2026-04-29

**Authors:** Emily Lunny, Helen Gray, Elen Davies, Amy Brown, Catrin Griffiths

**Affiliations:** ^1^ Swansea University Swansea Wales UK; ^2^ London School of Hygiene and Tropical Medicine London England UK

## Abstract

Breastfeeding is important for infant and maternal physical and mental health. Despite this, the United Kingdom (UK) and Ireland have the lowest breastfeeding rates in the world with between 34% and 52% of women breastfeeding partially or exclusively at 6–8 weeks across the nations. This is driven by complex biological, social, psychological and economic factors. However, a significant body of evidence shows that mothers who receive skilled breastfeeding support are more likely to breastfeed for longer. Effective breastfeeding support can be delivered by a range of trained professionals and peer supporters depending on need. The highest specialist support is provided by International Board Certified Lactation Consultants (IBCLCs). Research from the USA has shown the positive impact of IBCLC support upon breastfeeding duration and experience. However, there is limited data on this topic from the UK and Ireland. Given significant differences in IBCLC access and health care systems, this review therefore aimed to explore the impact of IBCLCs in the UK and Ireland. Of 5169 papers retrieved, only four studies met the eligibility criteria. Four themes were identified; breast milk feeding rates increased, breastfeeding duration increased, lack of specialised IBCLC support available outside of study and format of support delivery, including group based or 1‐1 support. The findings show increased access to IBCLC support may increase breastfeeding rates in the UK and Ireland. However, the findings are limited due to poor quality studies and recruitment bias. The paucity of evidence highlights the need for further research on this topic.

## Summary

- There is limited published evidence on the impact or outcomes of IBCLC support in the United Kingdom and Ireland, despite established evidence base elsewhere.
- IBCLC support in the UK and Ireland is associated with increased breastfeeding duration and increased rates of mothers' own milk feeding.
- Access to IBCLC support in the United Kingdom and Ireland was limited, as IBCLCs are not often available within universal healthcare services.

## 1 Introduction

It is well established that human milk is the optimal nutrition for infants, with other types of feeding associated with an increased risk of Sudden Infant Death Syndrome (SIDS), gastrointestinal disease and infections (Victora et al. 2016) and necrotising enterocolitis (Sami et al. 2023). For mothers, not breastfeeding is associated with an increased risk of breast cancer, type 2 diabetes and ovarian cancer (Victora et al. 2016). Breastfeeding can also play a significant role in maternal mental health, offering a protective effect when women meet their goals, but leading to significant distress when they cannot (Brown 2018).

However, despite the World Health Organization (2023) recommending breastfeeding for 2 years and beyond, the UK and Ireland have the lowest breastfeeding rates in the world (Victora et al. 2016). Although rates are slowly rising, the most recent routinely collected data for rates of any breastfeeding (partial or exclusive at 6–8 weeks are 52% in England (Office for Health Improvement and Disparities 2024), 51% in Scotland (Public Health Scotland 2025), 44% in Wales (Welsh Government 2025), 34% in Northern Ireland (Public Health Agency 2024. In Ireland, 42% of infants at 3 months are receiving any breastmilk (Health Service Executive 2024).

These figures do not always represent intention and are instead driven by a multigenerational shift to formula feeding practices, low investment in breastfeeding support, poor knowledge around how breastfeeding works, a lack of broader practical and emotional support for new mothers, return to work, and an environment of misinformation and predatory corporate marketing (Conway et al. 2023; Brown 2017).

To breastfeed successfully many mothers benefit from evidence based, skilled support outside or their family and peers (Gavine et al. 2022; Pérez‐Escamilla et al. 2023). Breastfeeding support in the UK and Ireland is often provided by a range of different health professionals and peer supporters. Healthcare practitioners who provide support include midwives, health visitors or public health nurses and doctors. While midwives often support mothers antenatally and in the early postnatal period, health visitors support mothers during early childhood with doctors typically only supporting breastfeeding during maternal or infant acute or chronic health challenges (WBTi 2025). Peer supporters (mothers with lived breastfeeding experience and around 35 h of training (ABM 2024)) are another source of breastfeeding support i.e. often available, and breastfeeding counsellors (including La Leche League Leaders) who have lived breastfeeding experience and around 18 months to 2 years of training (LLLGB 2022). Finally, IBCLC certification is the highest qualification in breastfeeding support (DeCoste et al. 2024). IBCLCs who must follow one of three training pathways, all requiring up to 1000 h of supervised experience, 14 health science modules at university level, a minimum of 95 h of breastfeeding specific education and a 4 h final examination (IBCLC Commission 2017).

There are currently 967 IBCLCs in the UK (IBLCE 2025) with 591,076 annual live births (Office for National Statistics 2023), this equates to a ratio of 1:611 IBCLCs to live infants born in the UK annually. In Ireland, there are 476 IBCLCs (IBLCE 2025) however with only 54,062 births (Central Statistics Office 2025), this equates to a ratio of 1:114 IBCLCs to infants born in Ireland. In the US, where IBCLCs are more widely embedded into healthcare systems, there are 20,539 IBCLCs (IBLCE 2025) with 3,596,017 annual births (National Centre for Health Statistics 2024). This equates to a ratio of 1:175 IBCLCs to infants born in the US annually. This data presents the stark differences between the number of IBCLCs practicing in the UK compared to the US.

In the US, IBCLC support is often covered by health insurance, or alternatively through organisations such as Women, Infants and Children (WIC) (Office on Women's Health 2021). The US private healthcare system is different to the NHS in the UK which offers free at the point of use care. In the U.S., there is a range of evidence showing improved breastfeeding outcomes when IBCLC support is provided (D'Hollander et al. 2025). A systematic review by Chetwynd et al. (2019) found postnatal IBCLC intervention may potentially improve breastfeeding outcomes.

However, IBCLCs are often less accessible in the UK due to lower rates of IBCLC employment in the NHS, with many IBCLCs working in private practice due to the qualification being unrecognised by some NHS trusts (Lopez‐Bassols et al. 2021). Overall, there is a lack of research investigating the impact of IBCLC support in the UK, and no scoping review currently exists which explores this topic.

This scoping review aims to explore the impact of IBCLCs on breastfeeding outcomes in the United Kingdom. Due to a lack of published research involving IBCLCs in the UK, this scoping review was extended to include both the UK and Ireland.

The purpose of this scoping review is to:

1. Establish an understanding of the impact of IBCLC support in the UK and Ireland.
2. Consolidate the current research and understanding relating to IBCLC support in the UK and Ireland and identify any impacts, weaknesses and strengths.

The following research question was identified to focus the review on IBCLC‐specific support and intervention in the UK: Does IBCLC support impact breastfeeding outcomes in the United Kingdom and Ireland, and what factors influence this?”.

## 2 Methods

This scoping review was conducted and reported in line with the PRISMA‐ScR checklist for reporting scoping reviews (Tricco et al. 2018). A published review protocol for this scoping review does not exist.

### 2.1 Eligibility Criteria

No time limit for the publication date of included studies was identified, due to an expected small number of eligible articles. Inclusion criteria restricted studies to those carried out in the United Kingdom (UK), expanding to include Ireland due to a distinct lack of relevant research available in the UK. Ireland was chosen due to similarities within society and the healthcare system to the UK (Connolly et al. 2022), compared to other countries with more available IBCLC research including the US (Haase et al. 2019). Studies which only explored antenatal interventions without postnatal support were excluded due to evidence that antenatal education alone is not likely to impact breastfeeding rates postnatally (Lumbiganon et al. 2016). Due to the small number of identified studies, both search engine and citation searches were also conducted to search for further relevant studies.

Eligibility criteria were reviewed and approved by the research team members (who are experts in breastfeeding research or IBCLCs) prior to the literature searches commencing.

### 2.2 Information Sources

Databases that were searched were: CINAHL (via EBSCOhost), Medline (via EBSCOhost) and Scopus (via Elsevier). Literature searches took place in February 2025.

All databases were searched without time limits due to the small amount of published research in this area.

### 2.3 Search

**Figure 1:**
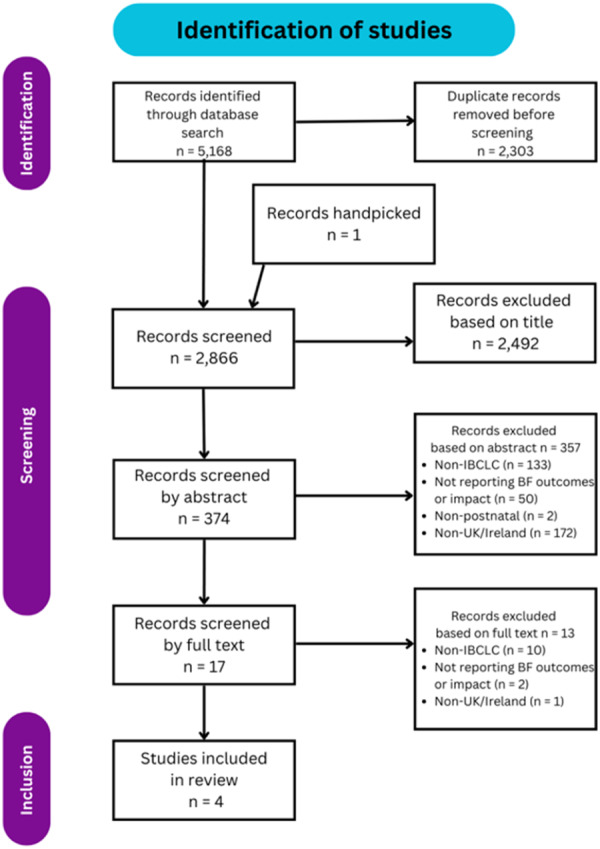
PRISMA flowchart describing article screening process.

Key terms (Figure 1) were identified to address the research question based on the Population, Exposure, Outcome (PEO) framework (Fineout‐Overholt and Johnston 2005). The PEO framework was chosen over others such as PICO (Population, Intervention, Comparator, Outcome) and PCC (Population, Concept, Context) as the PEO framework exposure component allowed for more reliable and relevant searching due to the need to identify studies utilising IBCLCs rather than any breastfeeding supporter who may not be an IBCLC.

Search terms and synonyms were identified (Table 1) and databases were searched using the identified terms in combination using Boolean operators. This included (Breastfeed* OR “Breast feed*” OR Breastfed OR Postnatal* Lactat* OR Infant feed* OR “Human milk” OR Breastmilk OR “breast milk”) AND (IBCLC OR “International Board Certified Lactation Consultant*” OR “Lactation Consultant*” OR “lactation support*” OR “breastfeed* support*”) AND (Perception* OR Perspective* OR Impact* OR Attitude* OR Opinion* OR View* OR Outcome* OR Satisf* OR Rate*), with each group referring to the population, exposure and outcome respectively (Tables 2, 3, 4).

**Table 1:** Inclusion and exclusion criteria.

Inclusion criteria	Studies written in the English language. Studies that focus on or include qualified International Board Certified Lactation Consultants. UK or Ireland‐based studies. Studies reporting breastfeeding outcomes or impact. Studies include postnatal support or intervention.
Exclusion criteria	Studies written in a non‐English language. Studies that did not include qualified International Board Certified Lactation Consultants. Studies based outside of the UK or Ireland. Studies that did not report breastfeeding outcomes or impact. Studies that only included non‐postnatal intervention e.g. antenatal only.

**Table 2:** PEO framework.

	Population	Exposure	Outcome
Population, Exposure, Outcome (PEO) term	Breastfeeding mother*	IBCLC or Lactation Consultant*	Breastfeeding outcomes
Synonyms	Breastfeed* Breastfeeding parent* Breastfeeding family* Postnatal* Lactat* Infant feed* “Human milk” Breastmilk “Breast milk”	“lactation support*” “breastfeed* support*” “International Board Certified Lactation Consultant*”	Perception* Perspective* Impact* Attitude* Opinion* View* Outcome* Satisf* Rate*

*Note:* Truncation terms are identified by an asterisk (*).

**Table 3:** Effective Public Health Practice Project (EPHPP) quality assessment—global ratings (McMaster Evidence Review & Synthesis Team 2022).

EPHPP global rating	Alberdi et al. (2018)	Conboy and Chacko (2023)	Lopez‐Bassols et al. (2021)	McGuinness et al. (2020)
Section A: Selection bias	3	2	3	3
1. Strong				
2. Moderate				
3. Weak				
Section B: Study design	3	2	3	3
4. Strong				
5. Moderate				
6. Weak				
Section C: Confounders	3	3	3	3
7. Strong				
8. Moderate				
9. Weak				
Section D: Blinding	3	2	2	3
10. Strong				
11. Moderate				
12. Weak				
Section E: Data collection methods	3	3	3	3
13. Strong				
14. Moderate				
15. Weak				
Section F: Withdrawals and drop‐outs	3	2	2	1
16. Strong				
17. Moderate				
18. Weak				
Global rating for this paper by both reviewers	3	3	3	3
19. Strong (no weak ratings)				
20. Moderate (one weak rating)				
21. Weak (two or more weak ratings)				
Is there a discrepancy between the two reviewers with respect to component (A‐F) ratings?	No	Yes	No	Yes
No				
Yes				
If yes, indicate the reason for discrepancy		2		2
22. Oversight				
23. Differences in interpretation of criteria				
24. Differences in interpretation of study				
Final decision of both reviewers	**3**	**3**	**3**	**3**
25. Strong				
26. Moderate				
27. Weak				

**Table 4:** Summary of themes.

Study	Theme				
	1: Breast milk feeding rates increased	2: Breastfeeding duration increased	3: IBCLC support not accessible outside of study intervention	4: The impact of the method of breastfeeding support	
				4a: Group breast‐feeding support	4b: 1‐1 breast‐feeding support
Alberdi et al. (2018)	Exclusive breast milk at 6 weeks 70%–73%; higher than general population.	Many women felt they breastfed for longer due to the intervention. Exclusive breast milk at 3 months 71.1%–60%; higher than general population.	Lack of access to IBCLC or skilled support outside of study.	Rural women preferred the breastfeeding group.	60% NMH/40% WGH found 1‐1 consult in hospital was most useful part.
Conboy and Chacko (2023)	Any breast milk at discharge increased from 59% (pre) to 85% (post/2021). Formula feeding on discharge 41% (pre) to 15% (post/2021). Earlier first assistance with hand expressing. 13% of infants had colostrum within 6 h 2019 (pre)—92% of infants had colostrum within 6 h (post/2021).		Lack of access to IBCLC/skilled support pre‐intervention.		
Lopez‐Bassols et al. (2021)	Of participants who's main concern was transitioning to exclusive breastfeeding, 100% were receiving mothers own milk beyond 6 months of age.	High percentage receiving mothers' own milk at 6 m (83%).	IBCLCs findings are different from primary presenting issues 75% of time. Poor NHS access to skilled support outside of intervention. Many participants had received poor or conflicting advice previously from other support. Issue solved by IBCLC 80% of the time, despite referral process requiring multiple previous support attempts.		One‐to‐one IBCLC consultations facilitated above average breastfeeding rates, with 83% of infants receiving mothers' own milk at 6 months of age, despite facing breastfeeding challenges. Examinations offered only possible in 1‐1 in‐person consultation.
McGuinness et al. (2020)		Increased breastfeeding rates—99% at 6 weeks, 92% at 3 months, 77% at 6 months.	Increased IBCLC access is needed.	Group support was highly valued.	Individual IBCLC was highly valued.

*Note:* Search limits were also placed to identify only English‐language and peer‐reviewed studies.

### 2.4 Data Extraction

A data extraction form was created to extract data from the included papers which included: author names, study design, results, strengths, limitations, sample size, location and themes (Appendix A). Data extraction was carried out by the primary author only due to time constraints; however extracted data was reviewed by two other authors. Data was charted by mapping themes to different categories, with themes identified inductively to avoid pre‐conceived theories in an under‐researched area.

### 2.5 Critical Appraisal

Included studies were appraised using the Effective Public Health Practice Project (EPHPP) (McMaster Evidence Review & Synthesis Team 2022) quality assessment tool by both the primary author and independently appraised by a second reviewer. The purpose of the critical appraisal was to systematically identify strengths, weaknesses and potential bias in the included studies. This quality assessment tool was chosen as it was appropriate for all the design of all four included studies. The CASP checklists (Critical Appraisal Skills Programme 2024) were considered but did not have a suitable option for retrospective audits.

## 3 Results

A total of 5168 published papers were identified through database searching. A total of 2303 of these records were found to be duplicates and removed before screening. One additional study was identified through reference list searches. After removal of duplicate studies, the remaining 2866 records were screened by title for relevance, retaining any potentially relevant papers for abstract review. A total of 2492 papers were found to be irrelevant by title, with the abstracts of 374 papers read. This stage was completed using EndNote by the primary author only, due to this study being part of a PhD.

A total of 357 papers were excluded based on abstract, including 172 excluded for being identified as outside of the United Kingdom or Ireland, 133 excluded for not including IBCLCs in the study, 50 excluded for not reporting outcomes and 2 excluded for only providing antenatal intervention.

Seventeen papers were retained for full text review by both the primary author and independently screened for eligibility by a second reviewer. Thirteen papers were excluded, with 10 excluded for not including IBCLCs. Full exclusion reasons are listed in Figure 1.

Four papers met the full eligibility criteria and were included in this scoping review.

### 3.1 Study Characteristics

The four included studies were published between 2018 and 2023 (Alberdi et al. 2018; Conboy and Chacko 2023; Lopez‐Bassols et al. 2021; McGuinness et al. 2020). Same sizes ranged between 75 (Lopez‐Bassols et al. 2021) and 211 (Conboy and Chacko 2023). The combined sample size of the four studies was 512 participants.

### 3.2 Study Location

Three studies were located in Ireland (Alberdi et al. 2018; Conboy and Chacko 2023; McGuinness et al. 2020) and one in London, United Kingdom (Lopez‐Bassols et al. 2021).

### 3.3 Study Design

All four included studies looked at outcomes of IBCLC intervention, with two studies comprising of a retrospective audit of existing service data using patient notes on breastfeeding outcomes (Lopez‐Bassols et al. 2021; Conboy and Chacko 2023), one cohort study with two groups receiving an intervention in different locations where participants received questionnaires at multiple timepoints to collect data (Alberdi et al. 2018) and one service evaluation where participants were telephoned to take part at random to provide service feedback and breastfeeding outcomes (McGuinness et al. 2020).

### 3.4 Outcome Measures

All four studies collected quantitative data, while McGuinness et al. (2020) was the only eligible study to also include qualitative data, which provided more detailed feedback about the intervention. Conboy and Chacko (2023) and Lopez‐Bassols et al. (2021) both collected data via audit. Alberdi et al. (2018) collected data from participants through questionnaires conducted antenatally, at 6 weeks and 3 months postnatal. McGuinness et al. (2020) collected all data via telephone in the calendar year after attendance, however it is unclear how recently some participants may have attended the intervention.

Alberdi et al. (2018), Conboy and Chacko (2023) and Lopez‐Bassols et al. (2021) all collected data on breastfeeding continuation rates. Alberdi et al. (2018) also collected quantitative data on uptake rates of offered interventions and ratings of which interventions were favoured by participants. Lopez‐Bassols et al. (2021) also collected data on whether IBCLC findings differed from previous health care professionals' findings or mothers' own concerns. McGuinness et al. (2020) also collected data on number of breastfeeding issues on presentation to the clinic. Conboy and Chacko (2023) collected data on timing of hand expression, timing of colostrum received and feeding method on discharge from NICU, before, during and after the intervention.

### 3.5 Intervention Format

Alberdi et al. (2018) conducted an intervention consisting of an antenatal breastfeeding class, followed by postnatal access to a one‐to‐one IBCLC consultation, motivational emails, a breastfeeding support group and a helpline. This was delivered in two comparator locations in Ireland: one rural and one urban.

Conboy and Chacko (2023) conducted two retrospective audits in a NICU, collecting results during two time periods—before recruiting one full‐time equivalent IBCLC and after IBCLC recruitment. Lopez‐Bassols et al. (2021) conducted a retrospective audit of all 75 service users who attended an NHS specialist outpatient referral‐only IBCLC clinic in London, United Kingdom. McGuinness et al. (2020) offered an IBCLC one‐to‐one clinic within a breastfeeding support group setting which could be attended for up to 6 weeks.

### 3.6 Overview of Individual Studies

Alberdi et al. (2018) included 127 participants, in addition to at least one support partner per participant. Only 100 completed the first stage of the intervention and 60 participants completed all data collection. Outcomes were measured by unvalidated quantitative questionnaires created by the authors, at 6 weeks and 3 months postpartum, with questions exploring participants' ratings of each study component, ratings on a Likert scale about which intervention components were perceived as most useful by participants and measurement of breastfeeding outcomes. Statistical analysis was described to be completed; however full results of the statistical analysis are not recorded in the paper.

Alberdi et al. (2018) results showed participants found the intervention useful and consistently rated the one‐to‐one IBCLC consultation valuable. Breastfeeding continuation rates after intervention were 60% (rural) and 70% (urban) at 3 months which is significantly above the Irish general population, where only 35% of babies are receiving breastmilk at 3 months (Purdy et al. 2017). However, the participants were self‐selected, therefore participants were more likely to be interested in breastfeeding and recruitment bias is likely to exist. Data may not be reliable also due to the significant number of participants lost to follow‐up (*n* = 67, 52.8% of participants). The strengths of this study included having two comparable locations where the intervention simultaneously took place, characteristics and demographics of participants were clearly identified and no conflicts of interest identified.

Conboy and Chacko (2023) conducted two retrospective audits using existing quantitative data from a total of 211 neonates at < 32 weeks gestation which were admitted to one NICU in Ireland during two time periods, in 2019 and 2020/2021. The first audit examined rates of breastfeeding, type of feeding on discharge and rates of any breast milk feeding in all 156 eligible admitted neonates before and after intervention, which was the employment of one full‐time equivalent IBCLC in October 2019. The first audit found an improvement, with rates of any breast milk feeding on discharge rising from 59% to 85%. The second audit consisted of a convenience sample of 61 infants with the same eligibility criteria as the first audit, with retrospective data collected from the same time period as the first audit. The second audit collected data on the timing of mothers' colostrum being available in the NICU and how quickly mothers were supported to hand express after birth, this audit found an increase from 13% to 92% of infants with colostrum available by 6 h after birth.

The strengths of the study by Conboy and Chacko (2023) were the inclusion of all < 32‐week infants admitted to the NICU for the first audit, reducing recruitment bias. The limitations included no available data on the demographics of participants, study authors are IBCLCs who may have a vested interest in proving positive outcomes associated with IBCLC intervention, no statistical analysis was included, and the timing of the recorded data means outcomes may have been influenced by Covid‐19 service changes, with the pre interventional data recorded from 2019 and the intervention data from 2020 to 2021 (Appendix A).

The study by Lopez‐Bassols et al. (2021) recorded quantitative data on breastfeeding duration, feeding type at 6 months and whether mothers' concerns aligned with IBCLC findings, for mothers attending their NHS referral‐only clinic between September 2018 and August 2019. The results found that IBCLC findings were often differing from the mothers' presenting concern. Lopez‐Bassols et al. (2021) also found 83% of participants were still breastfeeding at 6 months. Data is described as having undergone statistical analysis without detail of what type of analysis or the results of this. The strengths of this study were that it included an overall evaluation of area demographics, identification of the training level of those carrying out the intervention, with all being qualified IBCLCs and the use of an evidence‐based assessment tool when assessing lingual frenulums (Hazelbaker 2017). The limitations of this study included the restriction of individualised participant demographics data to age and ethnicity. There is no recorded data on other lactation or breastfeeding support encountered by the participants. Although it is mentioned that as the clinic is a referral service, participants have already received other breastfeeding support and intervention (Appendix A).

McGuinness et al. (2020) conducted a service evaluation recruiting women chosen at random who had attended their group‐based clinic. They collected data through a telephone questionnaire which recorded breastfeeding outcomes including duration of breastfeeding, parity and number of breastfeeding problems on presentation to the clinic in addition to qualitative data where women were asked about their experiences at the clinic. Results showed an increased breastfeeding duration compared to the general Irish population with 77% of participants still breastfeeding at 6 months. Qualitative feedback indicated the IBCLC clinic was highly valued, expertise was inaccessible outside of the study and group support was also beneficial. Data was described as analysed using a descriptive analysis for quantitative data and thematic analysis for the qualitative data. The strengths of the study were that no identified conflicts of interest and identification of training level of those carrying out the intervention who were all IBCLCs. Limitations included a non‐tested telephone questionnaire with no details on who delivered the questionnaire and if this may have included bias. Also, a lack of demographics data was recorded, except participants' nationality and an anecdotal observation by the researchers who found participants were mainly in professional employment and > 30 years of age, both of which are known to be associated with increased breastfeeding rates (Peregrino et al. 2018) (Appendix A).

### 3.7 Critical Appraisal

Quality assessment was conducted using the EPHPP quality assessment tool (McMaster Evidence Review & Synthesis Team 2022). Quality assessment was conducted independently by two reviewers (EL & HG). Quality assessment results were slightly different for two studies for example, within the category of study design, however after discussion with both reviewers and on re‐evaluation with the EPHPP guidelines final agreed results were agreed upon. Overall, all studies were rated weak during the quality assessment. However, it was noted that the existence of these studies in the context of a significant lack of relevant research in this subject area is positive and promising, despite methodical and selection bias concerns.

#### 3.7.1 Selection Bias

The main weakness was that three of four studies included potentially significant selection bias, with many participants likely to have been interested in breastfeeding whether they had participated in the study or not. This led to three studies (Alberdi et al. 2018; Lopez‐Bassols et al. 2021; McGuinness et al. 2020) receiving weak ratings. Conboy and Chacko (2023) received a moderate rating as all eligible infants were included in the audit.

#### 3.7.2 Study Design

All four eligible studies explored breastfeeding outcomes after IBCLC intervention. Two studies (Lopez‐Bassols et al. 2021; Conboy and Chacko 2023) consisted of retrospective service evaluations, however Conboy and Chacko (2023) received a higher rating of moderate due to including all babies at the specified gestation, whereas Lopez‐Bassols et al. (2021) study design meant that only those already breastfeeding were included. Alberdi et al. (2018) conducted a cohort study in two comparator locations where self‐selecting participants were offered a range of interventions, including IBCLC consultations and antenatal courses which corresponds with a weak rating. McGuinness et al. (2020) conducted a telephone audit collecting both quantitative and qualitative data from participants who had previously attended the IBCLC clinic within a group intervention. This corresponded with a weak rating.

#### 3.7.3 Confounders

All four papers received weak ratings in this category, as confounders (including maternal age, demographics and employment status) were present or not recorded and not controlled.

#### 3.7.4 Blinding

None of the participants for any of the studies were blinded to intervention, however with the selected study designs blinding is not expected therefore this section is not relevant. Conboy and Chacko (2023) and Lopez‐Bassols et al. (2021) both conducted service audits which correspond with moderate ratings. Participants of studies conducted by Alberdi et al. (2018) and McGuinness et al. (2020) self‐selected to take part in the intervention, so these papers received weak ratings. Although this is to be expected in psychological studies where it is difficult to blind participants to interventions.

#### 3.7.5 Data Collection Methods

Despite all four studies collecting quantitative data, and some describing having completed statistical analysis (Alberdi et al. 2018; Lopez‐Bassols et al. 2021), none of the papers included detailed results of statistical analysis. McGuinness et al. (2020) collected both quantitative and qualitative data using an unvalidated telephone questionnaire, which provided no detail as to who conducted the telephone questionnaire and whether this may have represented a bias. Alberdi et al. (2018) also used an untested questionnaire. Both Conboy and Chacko (2023) and Lopez‐Bassols et al. (2021) collected data from all eligible service users during a specified time period as part of service audits. All four studies received a weak rating for data collection methods.

#### 3.7.6 Withdrawals

Conboy and Chacko (2023) and Lopez‐Bassols et al. (2021) conducted service audits, which correspond with a moderate rating using the EPHPP. McGuinness et al. (2020) appears to have gained consent to participate in data collection from all participants contacted, achieving a strong rating. Alberdi et al. (2018) experienced a significant rate of withdrawals, with only 55% of participants at the rural location and 45% of participants at the urban location completing data collection, which corresponds with a weak rating.

### 3.8 Summary of Themes

Four themes and two sub‐themes were identified from the four included studies in relation to the research question which is “Does IBCLC support impact breastfeeding outcomes in the United Kingdom and Ireland, and what factors influence this?”. Three of the studies identified all four themes, with one study identifying two out of four themes. The first theme was increased breast milk feeding rates, which were noted in three out of four studies. The second theme was an increase in breastfeeding duration, which was identified in three out of four studies. The only study which did not identify the second theme had a study design which meant this thematic area was not explored. The third theme was a lack of skilled support or IBCLC support accessible or available outside of the study, which was identified in all four studies. The fourth theme was around the format of the support and was split into two sub themes; IBCLC led group support and IBCLC one‐to‐one support. The fourth theme was identified in three studies, but like the second theme this area was not explored in one study due to the study design.

### 3.9 Theme 1: Breast Milk Feeding Rates Increased

Three out of four studies reported increased rates of breast milk feeding (Alberdi et al. 2018; Conboy and Chacko 2023; Lopez‐Bassols et al. 2021). Alberdi et al. (2018) found that their intervention of an antenatal breastfeeding class, one‐to‐one IBCLC support postnatally and access to a support group, motivational emails and a helpline increased breast milk feeding rates, with 100% of participants who attended the antenatal course initiating breastfeeding. Similarly, of Lopez‐Bassols et al. (2021) participants whose main concern was transitioning to exclusive breastfeeding, 100% were receiving mothers' own milk beyond 6 months of age.

Contrastingly, the study by Conboy and Chacko (2023) also found increased rates of breast milk feeding. In 2019, the pre‐intervention group, the rate of any breast milk feeding at discharge was 59%, this increased during the intervention to 75% in 2020 and 85% in 2021. Exclusive breastmilk feeding rates also increased during this time, from 48% in 2019 pre‐intervention to 56% in 2020 and 79% in 2021. The rates of infants with their mothers' colostrum available within 6 h of birth increased significantly during the intervention period, from 13% in 2019 to 92% in 2021.

### 3.10 Theme 2: Breastfeeding Duration Increased

Increased breastfeeding duration was identified by three of the four included studies (Alberdi et al. 2018; Lopez‐Bassols et al. 2021; McGuinness et al. 2020).

Two of the included studies (McGuinness et al. 2020; Alberdi et al. 2018) which were based in Ireland offered one‐to‐one IBCLC support and a breastfeeding support group, with Alberdi et al. (2018) also offering email and telephone support. Alberdi et al. (2018) found that at 6 weeks postpartum, 50% of participants at the urban site and 73.3% of participants at the rural site reported they had breastfed for longer than they would have if they were not taking part in the study. At 3 months postpartum, 42.2% of participants at the urban site and 86.7% of participants at the rural site felt they had breastfed for longer than they would have without taking part in the study. McGuinness et al. (2020) also involved qualitative data collection; one participant who was breastfeeding at 15 months said *“[I] would have given up breastfeeding after a month.”* Both of these studies found increased breastfeeding duration. At 3 months postpartum, Alberdi et al. (2018) found rates of any breastfeeding at 70% in the urban site and 60% in the rural site while McGuinness et al. (2020) found breastfeeding rates of 91%. At 6 months postpartum, McGuinness et al. (2020) found breastfeeding rates of 77%. These 3‐month breastfeeding rates are an increase compared to the general population breastfeeding rates in Ireland of 35% at 3 months (Purdy et al. 2017).

These results were echoed by Lopez‐Bassols et al. (2021) who found that 83% of mothers attending the NHS specialist IBCLC clinic were providing their own breast milk to their infant beyond 6 months of age, which is significantly higher than the general UK population rate (which is 34% of 6 month old infants receiving any breast milk, NHS 2012). Lopez‐Bassols et al. (2021) found 76% of mothers who were referred to the clinic due to low milk supply were still providing breastmilk at 6 months postpartum. They also found 80% of mothers who were combination feeding but attended the clinic with the desire to move to exclusive breastfeeding had their issue resolved by receiving IBCLC care.

### 3.11 Theme 3: IBCLC Support Not Accessible Outside of Study Intervention

All four included studies identified a theme of a lack of skilled breastfeeding support available outside of the study. The study conducted by Alberdi et al. (2018) discusses how there are not enough lactation consultants in Ireland for every mother to have a consultation outside of the study, and the resulting lack of specialist support means 80% of mothers cease to breastfeed outside of the study.

McGuinness et al. (2020) conducted a study focussing on an IBCLC clinic also in Ireland; they found many of the clinic attendees were attending for further specialist support not provided elsewhere. This led to long waiting times for a one‐to‐one IBCLC consultation. One mother said, “*A lot of people and you just have to wait your turn.”* (McGuinness et al. 2020). They also mentioned how specialist support was even more important for the current generation as the previous generation had such low breastfeeding rates that family support could not be relied upon.

In another community‐based study, Lopez‐Bassols et al. (2021) discussed their local setting in the UK, where other breastfeeding support is provided by general practitioners, midwives and volunteer breastfeeding counsellors, and this support is often not adequate requiring referral to the specialist NHS clinic.

Conboy and Chacko (2023) conducted an inpatient, NICU‐based study and found prior to their intervention, there was no skilled breastfeeding support available in the NICU, and this was reflected in the significantly increased breastfeeding rates shown post‐intervention. The study concluded with a recommendation for other NICUs to recruit IBCLCs.

### 3.12 Theme 4: The Impact of Breastfeeding Support Format on Outcomes

Three studies identified key methods of IBCLC support format as positive (Alberdi et al. 2018; Lopez‐Bassols et al. 2021; McGuinness et al. 2020). Alberdi et al. 2018 offered participants one‐to‐one consultations alongside a support group, telephone and website support. Lopez‐Bassols et al. (2021) offered one‐to‐one IBCLC consultations. McGuinness et al. (2020) offered group‐based support with one‐to‐one sessions available during the group on a first come, first‐served basis. This shows that interventions, irrespective of the methods, can have an impact on breastfeeding outcomes.

#### 3.12.1 Theme 4a: Group Breastfeeding Support

Two studies offered group support as part of their intervention, and participants identified this as helpful (Alberdi et al. 2018; McGuinness et al. 2020). The study by Alberdi et al. (2018) found that group support was highly rated by participants, receiving 5/5 Likert scale score for both the urban and rural locations. 26.7% of participants at the rural location identified the breastfeeding support group as the most useful study component compared to 10% of participants at the urban location; however, due to the way this data collection is designed it is unclear why this support was favoured by some participants.

McGuinness et al. (2020) also conducted a study which involved participants attending a breastfeeding support group in an urban Irish location. Many participants enjoyed the group environment with feedback such as “*Other mothers chat was important.” “I made friends there.”* and that mothers were *“learning the skill together.”* However, multiple participants negatively noted the presence of men at the group, with one participant saying she felt a “little intimidated by males in the room” (McGuinness et al. 2020).

#### 3.12.2 Theme 4b: One‐to‐One Breastfeeding Support

Three studies reported the impact of one‐to‐one breastfeeding support (Alberdi et al. 2018; Lopez‐Bassols et al. 2021; McGuinness et al. 2020). Each study identified this theme in a different way due to differing study designs. Alberdi et al. (2018) found participants in all study locations scored the one‐to‐one IBCLC consultation an average 5/5 Likert scale score. 60% of participants at the urban location and 40% at the rural location voted the one‐to‐one IBCLC consultation the most useful component of the intervention. McGuinness et al. (2020) found one‐to‐one IBCLC support within their intervention was highly valued, with feedback such as “*Everything was good about the clinic, the one‐to‐ one time…weekly review was helpful.” and “The one‐to‐one is very important, as everyone is so different.”* They also found one‐to‐one support with the IBCLCs empowered women to succeed, with feedback including “*[name of the clinical midwife specialist] at the clinic was very motivating, she made me feel I could do it.”* Lopez‐Bassols et al. (2021) found that specialist one‐to‐one IBCLC consultations facilitated above average breastfeeding rates, with 83% of infants receiving mothers' own milk at 6 months of age, despite facing breastfeeding challenges. They also offered intraoral examinations to assess for ankyloglossia, at breast supplementation and other clinical skills which are only possible in one‐to‐one, in‐person consultations.

## 4 Discussion

This scoping review aimed to establish the impact and outcomes of IBCLC support in the United Kingdom (UK) and Ireland. There is a lack of research in this subject area in the UK, highlighted by this scoping review only finding four eligible studies, only one of which was based in the UK (Lopez‐Bassols et al. 2021). These four studies were all rated as weak during quality assessment, with data collection methods being a particular weakness in all studies, and recruitment bias a significant weakness in three studies (Alberdi et al. 2018; Lopez‐Bassols et al. 2021; McGuinness et al. 2020).

Four themes were identified. These themes were: increased breastmilk feeding rates, increased duration of breastfeeding, a lack of accessible, skilled support outside of the study and format of IBCLC support, which was split into two sub‐themes of group support and one‐to‐one support. None of the studies featured a comparative group. All studies measured breastfeeding outcomes without reporting psychological outcomes for women, however McGuinness et al. (2020) briefly mentioned success in empowering women to succeed through IBCLC support. The first two themes identified an impact upon breastfeeding initiation and continuation rates, while the following themes explored how this increase may have been facilitated.

### 4.1 Increased Breast Milk Feeding Rates

Any breast milk feeding is associated with increased positive health outcomes for infants. Victora et al. (2016) found a 36% reduction in SIDS deaths for babies ever breastfed. IBCLC support was found to be associated with increased breast milk feeding rates, with Conboy and Chacko (2023) reporting an increase in rates of any breastfeeding at NICU discharge as having increased from 59% pre‐intervention to 81% after intervention with IBCLC support available. Other studies have explored the introduction of an IBCLC into a NICU and the impact on rates of any breast milk feeding. Hoban et al. (2021) studied proactive IBCLC support in a NICU to reactive support, finding proactive IBCLC support was associated with an increase from 74.3% to 80.2% of any breast milk feeding, and a cohort increase in milk storage needed. A study into the differences in breast milk feeding rates in NICUs in England found a variation ranging from 48.6% to 79.3% of infants receiving any mother's own milk at discharge, with specialist infant feeding support being associated with higher rates (McLeish et al. 2024).

Conboy and Chacko (2023) also found a large increase in rates of infants who had their mother's colostrum available within 6 h of birth, which increased from 13% pre‐intervention to 92% after intervention. With a lack of recruitment bias in this study, these were the most reliable findings. Alberdi et al. (2018) and Lopez‐Bassols et al. (2021) both found universal breast milk feeding rates, with all participants breastfeeding. This is in contrast to Ireland's rates of 58% of infants receiving any breastmilk on discharge from hospital (Purdy et al. 2017) and the UK's breastfeeding rates of 81% of infants ever receiving any breast milk during the most recent national infant feeding survey (NHS 2012). However, Alberdi et al. (2018) and Lopez‐Bassols et al. (2021) study participants were self‐selecting.

### 4.2 Increased Breastfeeding Duration

Breastfeeding duration matters as health benefits for both mother and child increase with increased breastfeeding duration. This includes a reduced risk of breast and ovarian cancer for the mother (Stordal 2022). Up to 80% of women in the UK cease breastfeeding before they planned to (NHS 2012). The results from this scoping review are consistent with a recently published systematic review and meta‐analysis which found IBCLC interventions in high‐income countries increased breastfeeding duration, including duration of any breastfeeding (D'Hollander et al. 2025).

This scoping review found breastfeeding duration was increased compared to the general population. Lopez‐Bassols et al. (2021) found 84% of mothers who received IBCLC support were providing their own milk at 6 months postpartum. This is significantly higher than the UK general population, with 34% of infants receiving any breast milk during the last national infant feeding survey in 2010 (NHS 2012). Similarly, two Irish studies (Alberdi et al. 2018; McGuinness et al. 2020) found breastfeeding rates of 60%–70% and 91% respectively at 3 months post‐partum. This is a significant increase compared to the general population of Ireland, where rates of any breast milk feeding at 3 months are 35% (Purdy et al. 2017). Yourkavitch and Hall Smith (2022) found that each IBCLC per 1000 live births increased breastfeeding at 6 months by 5% and increased breastfeeding at 12 months by 4%, in their US‐based study which controlled women's demographics status.

### 4.3 Lack of Accessible Skilled Breastfeeding Support

The following themes identified how the increase in breastfeeding initiation and duration identified in previous themes may have occurred. While broader breastfeeding support in the form of peer support and health care professional access is available, a lack of accessible specialist IBCLC breastfeeding support outside of the study intervention was identified by all four studies. Compared to other countries such as the US, IBCLC support is significantly harder to access due to restrictions in the UK's NHS in employing IBCLCs (Lopez‐Bassols et al. 2021). The IBCLC credential is not formally recognised in the UK, meaning the qualification is not valued within many NHS trusts. This had led to IBCLCs often only able to access low‐paid NHS jobs whilst maintaining a costly qualification, and restricted from infant feeding lead roles, or choosing to enter private practice.

In both the UK and Ireland, IBCLCs in many areas are only accessible through private practice, with over 500 of the UK's 967 IBCLCs (IBLCE 2025) registered for private practice on the Lactation Consultants Great Britain website (LCGB 2025). This further perpetuates the health inequities faced by those with lower incomes, who already have lower rates of breastfeeding (Peregrino et al. 2018), are more likely to live in areas where formula feeding is the norm and can struggle to access breastfeeding support more broadly (Hunt et al. 2021). Breastfeeding support is often available from other practitioners and peer supporters; however, many professions receive inadequate training as part of their required pre‐registration education (WBTi 2025), leading to a variation in knowledge levels and skills amongst health care professionals who support breastfeeding (Norman et al. 2022).

A lack of skilled breastfeeding support outside of the intervention meant a large number of attendees were waiting at the weekly IBCLC clinic in the study by McGuinness et al. (2020). Conboy and Chacko (2023) made a recommendation to other NICUs to hire IBCLCs after seeing a significant increase in breastfeeding rates and availability of mothers' colostrum. Alberdi et al. (2018) discuss how there are not enough lactation consultants in Ireland to allow for each mother to have a consultation, resulting in a lack of specialist support leading to low breastfeeding rates.

### 4.4 Format of IBCLC Support

Breastfeeding support groups are evidenced to increase breastfeeding duration and self‐efficacy (Rodríguez‐Gallego et al. 2024) while one‐to‐one IBCLC support consultations are also known to increase breastfeeding rates and solve complex issues which are often unable to be solved by other practitioners (D'Hollander et al. 2023). A systematic review by Pascual et al. (2025) found face‐to‐face IBCLC support had a positive effect on exclusive breastfeeding rates at 6 months. Support can also be delivered by video call, telephone helplines, email, websites and apps which may be more accessible to some mothers but are not universally offered and often reliant on volunteers (Morse et al. 2025). During this scoping review, two types of support were positively identified as sub‐themes.

Group breastfeeding support was seen as beneficial by two studies in this scoping review (Alberdi et al. 2018; McGuinness et al. 2020) which included this type of support. This is consistent with previous research by McCarthy et al. (2024) who found that the majority of women who accessed a breastfeeding support group in Ireland were breastfeeding beyond 12 months, however this research identified this finding in breastfeeding counsellor‐led support groups, so may not be applicable to IBCLC led groups.

One‐to‐one IBCLC support was also positively identified by three studies. Lopez‐Bassols et al. (2021) found that one‐to‐one support increased breastfeeding rates at 6 months and the likelihood of breastfeeding issues being solved, even if other practitioners were previously unable to. Alberdi et al. (2018) offered many types of support during their intervention, with 60% of participants at the urban location voting one‐to‐one IBCLC support as the most useful study component. This finding is consistent with existing research by Otiv et al. (2024) who found 90.2% of mothers receiving one‐to‐one lactation consultant support in the early postpartum period were discharged from hospital exclusively breastfeeding, compared to 59.1% in the control group.

Overall, the findings of this scoping review show that for women who were already interested in or planning to breastfeed, IBCLC support in these studies likely contributed to increased breastfeeding. Despite a very limited amount of published research on this topic, IBCLC support shows potential to improve breastfeeding outcomes in the UK and Ireland.

### 4.5 Limitations

There was no registered protocol for this scoping review. Only four studies met the eligibility criteria due to a small number of relevant published papers in the geographic location identified. All four included studies were rated weak after quality assessment, with key limitations presenting due to study design, self‐selecting participants, lack of controlling for confounders and non‐validated data collection methods.

When studies recorded demographic data, it was identified that many participants were educated, employed and > 30 years old. These factors are independently known to be associated with increased breastfeeding rates (Peregrino et al. 2018). One study did not record any demographic data (Conboy and Chacko 2023) but included all admitted patients at the relevant gestational age and found a significant increase in breastfeeding rates and breast milk availability. As this study included all eligible infants, it removed the potential for recruitment bias, mitigating this limitation.

All included studies only measured breastfeeding outcomes in terms of breastfeeding initiation and duration. None of the included studies explored perinatal mental health, social, self‐efficacy or health outcomes, which future research could focus on.

The design of these studies meant that often only those already motivated to breastfeed were participants, as women typically sought out IBCLC themselves (Alberdi et al. 2018; Lopez‐Bassols et al. 2021; McGuinness et al. 2020). Motivation and determination to breastfeed is associated with increased breastfeeding duration (Kestler‐Peleg et al. 2015). IBCLC support has not yet been tested in a broader population of women, and future research should explore the impact of receiving IBCLC support when women are not highly motivated, knowledgeable and determined to breastfeed. Although it is an important finding that IBCLC support can help those who are motivated to breastfeed, it would be interesting to know whether it can help change perceptions, beliefs and motivation in those less positive to start with. Study design, which included two retrospective service evaluations, a telephone service evaluation and a cohort study, also meant results are less reliable compared to a randomised controlled trial. Undertaking an RCT would be valuable, offering IBCLC support in addition to standard care to test its efficacy and potentially expand to more women.

The results of this scoping review show that IBCLC support is likely to increase breastfeeding initiation and duration in the UK and Ireland. However, this finding is limited due to poor quality and potential recruitment bias in these studies. Further high‐quality research is warranted.

## 5 Conclusions

There is a very limited amount of published evidence on IBCLC support in the United Kingdom and Ireland. However, the current limited knowledge in this geographical location shows a positive impact of IBCLC support and breastfeeding outcomes. The results of the reviewed studies show for those planning to breastfeed, IBCLC support may have the potential to increase breastfeeding duration, breast milk feeding rates and provide high‐quality support. However, due to the poor quality of these studies and recruitment bias there is no evidence that these results are reliable for the general population in this location. Further research into this area is critically needed, which may evidence the need for further funding of specialised breastfeeding support to increase the exceptionally low breastfeeding rates in the UK and Ireland. Specific areas to focus on may include an intervention or randomised controlled trial of IBCLC support, understanding who currently accesses and might feel excluded from IBCLC support and how to expand this, and challenges integrating IBCLC support into standard care.

## Author Contributions

E.L. was responsible for study conception, database searching, selection and quality appraisal of all articles, data extraction, writing of the draft manuscript and critical revisions. H.G. was responsible for full‐text article selection and quality appraisal. E.D. was responsible for database searching and critical revisions. A.B. was responsible for study conception, review support, draft manuscript support and critical revisions. C.G. was responsible for study conception, review support, draft manuscript support and critical revisions.

## Conflicts of Interest

Two authors, E.L. and H.G. are IBCLCs. The authors declare no conflicts of interest.

## Appendix A Summary of Reviewed Articles

**Table:**

Study	Strengths	Limitations	Location	Sample size	Design	Population	Results
Alberdi et al. (2018)	Study conducted in two comparator locations, one rural and one urban. Characteristics of participants clearly identified. There were no conflicts of interest identified and study was funded by a non‐biased organisation.	Large percentage of participants did not complete data collection. Participants were already interested in breastfeeding which may cause results to be not applicable to the general population. Study participants were highly educated. One of the lactation consultants delivering the intervention had not yet qualified at the time of intervention.	Ireland	127	Cohort study	Two participant groups, one group from a rural hospital and one group from an urban hospital.	60% urban/40% rural found 1‐1 consult in hospital was most useful part. Rural women preferred the breastfeeding group and helpline. Many women felt they breastfed for longer due to the intervention. Breastfeeding support group and 1‐1 sessions were consistently rated most helpful Exclusive breast milk at 6 weeks 70%–73%; higher than general population. Exclusive breast milk at 3 months 71.1%–60%; higher than general population. Lack of access to IBCLC or skilled support outside of study.
Conboy and Chacko (2023)	Study includes breast milk feeding outcomes of all discharged NICU patients < 32 weeks gestation at birth, mitigating recruitment bias. Study identified training levels of those carrying out intervention, all were IBCLCs. There were no conflicts of interest identified and study was funded by a non‐biased organisation.	Participants demographics not recorded. Study authors are IBCLCs who may have a vested interest in proving improved outcomes with IBCLC intervention. Study results may be affected by COVID‐19 support changes, with control group data from 2019 and intervention group data from 2020 to 2021.	Ireland	211	Retrospective audit	Control group consisting of neonates < 32 weeks gestation at birth in relevant NICU pre‐IBCLC staff member and intervention group consisting of neonates < 32 weeks gestation at birth in relevant NICU post IBCLC recruitment.	Any breast milk at discharge increased from 59% (pre) to 85% (post/2021). Formula feeding on discharge 41% (pre) to 15% (post/2021). Earlier first assistance with hand expressing. 13% of infants had colostrum within 6 h 2019 (pre)—92% of infants had colostrum within 6 h (post/2021). Lack of access to IBCLC/skilled support pre intervention.
Lopez‐Bassols et al. (2021)	Area demographics data is included, but not applied individually to each participant. Assessment tools are clearly detailed. There were no conflicts of interest identified, and study was funded by a non‐biased organisation. Study identified training levels of those carrying out intervention, all were IBCLCs.	Individual demographics data is limited to ethnicity and age of mother and baby. Participants were already breastfeeding so may not be indicative of the general population who may not have initiated breastfeeding. Study does not record other lactation support received by participants but mentions they have often had numerous other interventions.	United Kingdom	75	Retrospective audit	All specialist clinic attendees between September 2018‐August 2019.	High percentage receiving mothers' own milk at 6 m (83%). Many participants had received poor or conflicting advice previously from other support. Issue solved by IBCLC 80% of the time, despite referral process requiring multiple previous support attempts. IBCLCs findings different from primary presenting issues 75% of time. 21.3% perceived low milk supply. Poor NHS IBCLC access.
McGuinness et al. (2020)	There were no conflicts of interest identified although it is not clear who funded the study. Study identified training levels of those carrying out intervention, all were IBCLCs.	No demographics data was collected from participants. Anecdotal information from researchers which found participants were majority in professional employment and > 30 years of age which is known to be associated with increased breastfeeding rates.	Ireland	99	Service evaluation	Clinic attendees who had attended during 2017.	Individual IBCLC was highly valued. Group support was highly valued. Increased breastfeeding rates—99% at 6 weeks, 92% at 3 months, 77% at 6 months. Participants felt empowered to breastfeed. 63% presented with one breastfeeding challenge, 24% with two breastfeeding challenges. Increased IBCLC access is needed.
